# Study on Nonlinear Conductivity of CCTO/EPDM Rubber Composites

**DOI:** 10.3390/ma11091590

**Published:** 2018-09-02

**Authors:** Zhongyuan Li, Hong Zhao, Changhai Zhang

**Affiliations:** 1 Key Laboratory of Engineering Dielectric and its Application, Ministry of Education, College of Electrical and Electronics Engineering, Harbin University of Science and Technology, Harbin 150040, China; zli_ma16@hrbust.edu.cn (Z.L.); hongzhao@hrbust.edu.cn (H.Z.); 2 College of Science, Harbin University of Science and Technology, Harbin 150040, China

**Keywords:** copper calcium titanate, nonlinear conduction, electric field simulation, breakdown

## Abstract

Researches of the theories and application of polymer composites with nonlinear conductivity are useful for dealing with the nonuniform electrical fields widely existing in the cable accessory insulation. In the present work, we fabricated CCTO (CaCu_3_Ti_4_O_12_)/EPDM (Ethylene Propylene Diene Monomer) composites and investigated their breakdown strength, dielectric and nonlinear conductivity properties in detail; the microstructures of fillers and composites were characterized by scanning electron microscopy (SEM) and X-ray diffraction. CCTO particles are uniformly dispersed in CCTO/EPDM composites, and the composites showed nonlinear conductivity with electric field changes. When the CCTO particle content is low, the conductivity of CCTO/EPDM composites does not present obvious nonlinearity. However, when CCTO content exceeds 2 vol %, the conductivity experiences a nonlinear change with increasing electric field strength and the threshold field (E_th_) of nonlinear conductivity declines with the increase of CCTO contents. In addition, it can be found from experiment and simulation results that 8 vol % CCTO/EPDM exhibit significant nonlinear conductivity and dielectric properties as expected, and homogenizing the electrical field much more effectively. Therefore, this paper offers a preliminary discussion about the variation trend of nonlinear conductivity CCTO/EDPM composites, providing an effective reference to solve the application of nonlinear conductivity materials for cable accessories.

## 1. Introduction

High voltage direct current (HVDC) transmission has many advantages such as low loss, large transmission capacity, high operational stability, low environmental effect, cost-effectiveness, and so on. With major breakthroughs in converter technology, its development has received widespread attention [1]. ABB (Asea Brown Boveri) announced the new 525 kV direct current (DC) cable system with a power rating range up to powers above 2 GW that has been developed for both subsea and underground applications [2]. The Prysmian Group declared successful development and testing of its new P-Laser 525 kV cable system for HVDC applications [3]. Cable accessories are an important part of power cable system. Cable accessory faults are the most common among many factors of power cable system faults. The statistics of power cable system faults show that cable accessory fault account for approximately 70% of total cable faults [4]. Therefore, cable accessories need to be carefully designed: as they are a weak link in the cable power system and make the distribution of the internal electric field more reasonable. For cable accessories, there are different types of interface structure, such as interface structure formed by XLPE (Cross-linked polyethylene) and enhanced insulation composites. Under DC voltage, a large amount of space charge is accumulated in the interface structure, and it causes serious distortion of the electric field distribution in cable accessories [5,6]. Reasonable design of the structure of and the optimization of their material properties have become prominent requirements for controlling the electric field.

Since the 1990s, scientists first proposed the concept of nano-dielectrics, and the exploration and research on nanocomposites has been a hot topic and advanced subject. The dielectric properties of a polymer can be significantly changed by adding nanofillers [7,8]. In order to homogenize the electric field and design reliable cable accessories with stable operation and high universality numerous scholars have concentrated on studying nonlinear dielectrics. The conductivity of nonlinear composites increases nonlinearly as electric field strength increases, which leads to the local area of material present and strong electric field strength, space charge will redistribute and local electrical field strength will reduce [9,10,11,12]. L.G. Virsberg et al. proposed a vision that the electric field can be improved using nonlinear dielectrics [13]. ABB researchers studied a technical scheme of cable sleeve that nonlinear composites could be substituted for high conductive composites with constant parameters; the results show that the electric field distribution of the cable sleeve is improved [2]. 

In a previous study, nonlinear composites were prepared by silicon carbide (SiC) or zinc oxide (ZnO) as nonlinear fillers. Testsushi Okamoto et al. found that SiC and Fe_3_O_4_ were fillers doping in polybutadiene, and the nonlinear characteristics of volt-ampere was determined by SiC; the nonlinear threshold of field strength increases as Fe_3_O_4_ concentration increases [14]. B.R.Varlow researched the DC voltage characteristics of ZnO/LDPE (Low Density Polyethylene) composites. When the concentration of ZnO exceeded 10 wt %, composites had obvious nonlinear volt-ampere characteristics. Moreover, the current density of 10 wt % ZnO/LDPE was lower than pure LDPE [15]. It has been recognized that strongly nonlinear effects in ZnO powders are due to the special structure of fillers: the core of the highly doped ZnO semiconductor is enclosed by a thin complex mixed metal oxide shell (grain boundary in bulk ceramic varistor) that is responsible for the nonlinearity [16,17]. The different crystal forms of SiC are also different nonlinear effects; nonlinearity occurs only when the content of SiC is large enough [18,19]. However, the excessive content may cause the deterioration of mechanical properties and breakdown strength. Therefore, the difficulty of preparing nonlinear composites also increases.

In order to adapt the need of higher voltage levels and multiple types of insulation, it is important to research new types of nonlinear composites, which solve the problem of uneven electric field distribution in insulation equipment. Especially for the development of technologies and industries, it has the function of filling a gap in basic theory and applied research, the value and significance of this are even more significant.

Copper Calcium Titanate (CCTO) has a perovskite structure and high dielecric constant at room temperature, so CCTO particles are mostly used for the preparation of high-dielectric composites [20,21]. There are few researches about the nonlinear conductivity of CCTO particles. In the present work, EPDM rubber was a matrix of composites; CCTO particles were prepared as fillers. The nonlinear conductance and dielectric properties of CCTO/EPDM composites were investigated in detail. The steady and transient electric field characteristics of CCTO/EPDM composites were investigated by COMSOL Multiphysics theoretical simulation, the feasibility of CCTO/EPDM composites as reinforced insulation was confirmed.

## 2. Materials and Methods

### 2.1. Raw Materials

CCTO particles were prepared by the sol-gel technique. Firstly, 21.744 g Cu(NO_3_)_2_·3H_2_O and 7.0845 g Ca(NO_3_)_2_·4H_2_O were put into a beaker with 160.0 mL C_3_H_8_O_2_, then 41.0 mL Ti(OC_4_H_9_)_4_ was slowly poured into the above solution, with stirring, for another 3 h; CCTO sol solutions were obtained. Secondly, the CCTO sol was allowed to stand at room temperature for 24 h to obtain gelatum. Finally, the CCTO gelatum was grinded into a powder, followed by sintering in a muffle furnace with a heating rate of 5 °C/min from 0–300 °C, keeping at 300 °C for 2 h, then, with the same heating rate, from 300–1050 °C, keeping at 1050 °C for another 8 h; the crystallized CCTO ceramics were then obtained. Finally, the CCTO ceramics were obtained by Planetary Ball Mill for 12 h with 450 rpm. The above materials are listed in Table 1.

This article uses two-roll milling and hot-pressing to prepare composites. EPDM was matrix, and the CCTO particles were nonlinear fillers; dicumyl peroxide (DCP) was employed as the cross-linking agent. The raw materials were mixed uniformly in a two-roll mill at 363 K for 30 min. Then the mixture was cured in a stainless steel mold at 433 K under a pressure of 15 MPa for 15 min, the samples had a thickness of 0.2 mm.

### 2.2. Performance Testing of Composites

The phase of composites was investigated by X-ray diffraction (XRD, Mpyren), which was purchased from PANalytical B.V, Almelo, Netherlands. The Cu target (wavelength of 1.5418 Å) was applied as the electron emission target. The working current was 30 mA and the operating voltage was 40 kV. Scan angle 2θ range was 10–100°, the scan rate was 4°/min. The dispersion of CCTO particles was observed by SEM, which were purchased from Hitachi Limited SU8020, Shanghai, China. 

**Figure 1:**
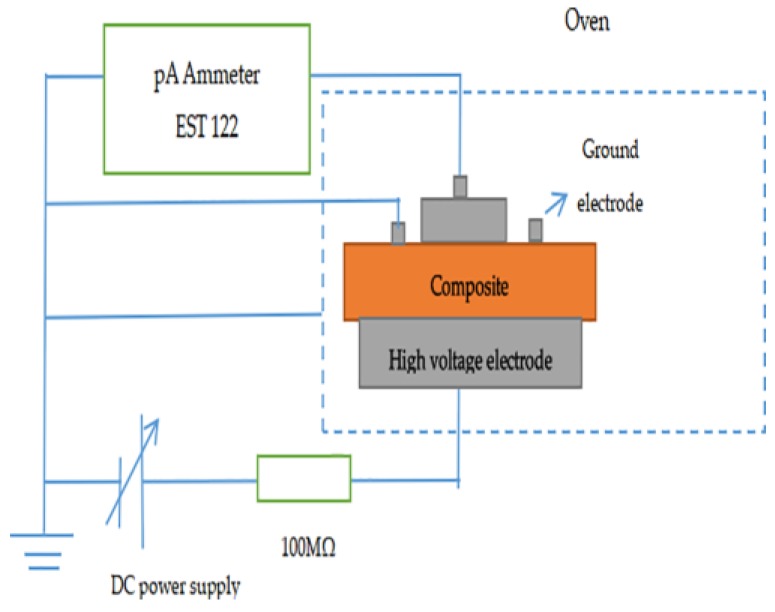
Three-electrode test system schematic.

A three-electrode system (Harbin University of Science and Technology, Harbin, China) was used for measuring DC volume conductivity of samples, and the thickness of samples was approximately 0.2 mm, the size was 10 × 10 cm^2^. The system was composed of a high-voltage DC power supply (continuously adjustable output voltage from 0 kV to 20 kV), an Ammeter EST122 (pA Ammeter test range of 20 × 10^−3^ A–1 × 10^−15^ A), and an oven (the maximum operating temperature was set to 200 °C), as shown in Figure 1. The sample was put into the oven then connected to the electrode and preheated for 2 h, after that, while increasing the DC power output voltage gradually, each voltage level keeping 30 min, then recording the current and the voltage value. In order to avoid accidental errors, we prepared three samples for each sample and then averaged the three measured results.

The dielectric properties of composites were measuring by Agilent 4294A Precision Impedance Analyzer (Agilent, Santa Clara, CA, USA), the thickness of samples was about 0.2 mm and the size was 30 × 30 mm^2^. The highest frequency resolution was 1 MHz. The upper and lower electrodes were both 20 mm, and the test condition was room temperature. The range of frequency was 10^0^ Hz–10^6^ Hz. Before measuring conductivity, the dielectric spectrum, and breakdown of composites, samples needed to be short-circuited at 80 °C, which prevents the result of experiments from being affected by moisture and residual charge inside the sample [22,23,24]. 

## 3. Results

### 3.1. Microstructures Characterization

**Figure 2:**
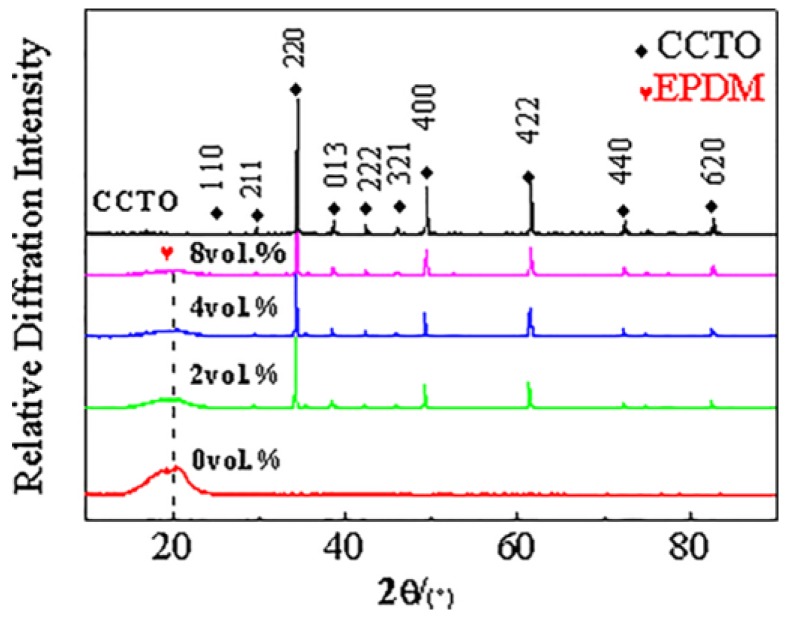
X-ray diffraction patterns of CaCu_3_Ti_4_O_12_ (CCTO) particles and CaCu_3_Ti_4_O_12_/ Ethylene Propylene Diene Monomer (CCTO/EPDM) composites.

The XRD spectra of different samples are shown in Figure 2. CCTO particles have a perovskite structure and no impurity peak. Compared to the results of the XRD spectra, it can be found that there is a certain difference in these spectra. Although, when CCTO particles are dispersed in the EPDM and do not find the phase transition of CCTO, the strength of the CCTO phase increases with increasing of content, this is because the intensity depends on the volume fraction of CCTO in the composite and its linear absorption coefficient. The neat EPDM have a broad diffraction peak at 2θ = 20°, but the diffraction peak of the EPDM phase is very weak in the CCTO/EPDM composites, and its intensity decreases with the increase of CCTO content. This is due to CCTO particles dispersed in the composite that may destroy the regular arrangement of the EPDM molecules, leading to the diffraction peak of neat EPDM become insignificant. 

**Figure 3:**
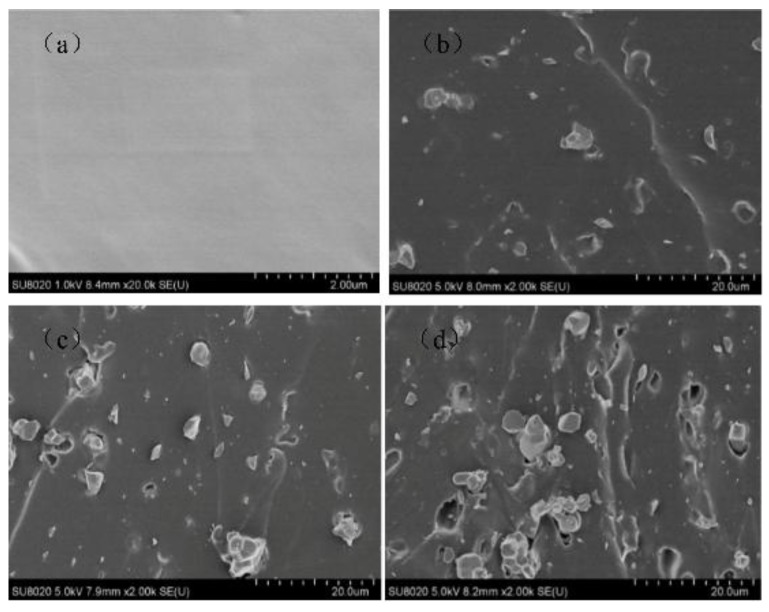
SEM morphology of the fresh surfaces of EPDM and composites with different CCTO contents: (**a**) pure EPDM; (**b**) 2 vol % CCTO/EPDM; (**c**) 4 vol % CCTO/EPDM; (**d**) 8 vol % CCTO/EDPM.

The cross-section of CCTO/EPDM composites was observed by scanning electron microscopy (SEM), as shown in Figure 3. It can be seen that the CCTO particles are uniformly dispersed in the EPDM matrix, and the size of most of the CCTO, as shown in Figure 3, is approximately 2–5 μm, a few particles are approximately 100–300 nm. There is no obvious defect; this indicates that CCTO particles have good contact with the EPDM rubber matrix.

### 3.2. The Conductivity of Composites 

#### 3.2.1. The Characteristic of Conductivity (Γ)—Electric Field (E)

The empirical formula for the relationship between electric field and conductivity is:

γ = αE^β^ (1)

In the Equation (1), β is the nonlinear conductivity coefficient of material, α is a constant. By logarithm Equation (1), Equation (1) is converted to linear form:

lgγ = lgα + βlgE(2)

**Figure 4:**
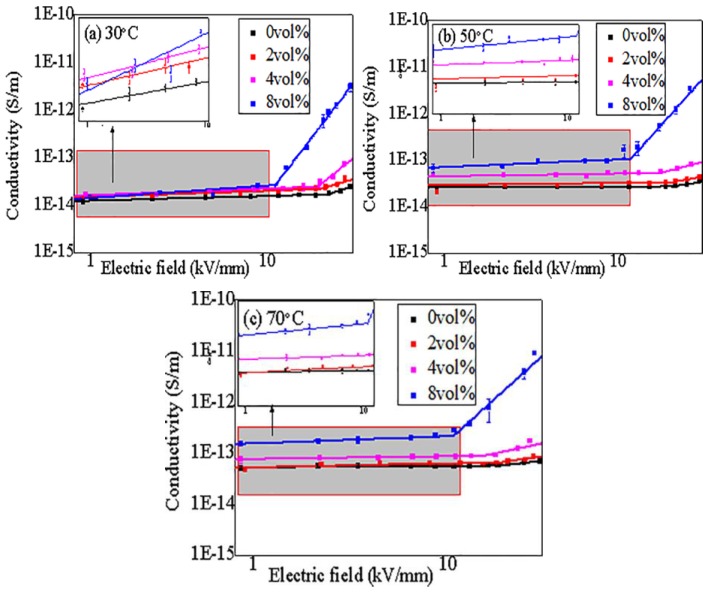
Conductive characteristics of composites filled with CCTO at different temperatures.

The value of the nonlinear coefficient β can be obtained by Equation (2). The conductivity mechanism of composites may change with applied electric field or temperature, which determines the different relationship between applied electric field (E) and the conductivity (γ) of CCTO/EPDM. The γ-E curve of CCTO/EPDM is shown in Figure 4 at 30, 50, and 70 °C. It can be seen that the γ-E curve of CCTO/EDPM has an inflection point when the electric field increases, this phenomenon can be explained by the SCLC (space-charge-limited-current) theory. Due to the discontinuous energy level in polymer, charge carriers are easily trapped by traps to form space charges under a lower electric field, so the conductivity of EPDM increases inconspicuously with the change of the electric field. When the applied electric field exceeds a certain threshold electric field (E_th_), the trapped carriers can obtain enough energy to become detrapped and participate in the conduction process, leading to a significant increase in conductivity. 

Dispersing CCTO particles will make the conductance mechanism more complex. Firstly, the conductivity characteristics of 2 vol % CCTO/EPDM display a similar trend to that of EPDM. According to the percolation theory [25], the average distance is enough large between particles under doping slight content of conductive or semiconductive particles, leading to charge carriers migrate difficultly between particles. Thus, doping low content of CCTO particles does not change the conduction mechanism of composites. Besides, it can be found that the conductivity of 2 vol % CCTO/EPDM is slightly higher than that of EPDM, the improvement in conductivity is shown to be due to the doping CCTO particles that make the change in chain structure in the polymer, where the stretched conformation of polymer chains changes [26,27,28]. Secondly, with the increase of doping content, when the applied electric field E < E_th_, carriers cannot go through the barrier between the CCTO and EPDM matrix. However, when E > E_th_, the tunneling effect occurs [29]. A large number of carriers can directly transport through trap barriers by tunneling in the high electric field area, and can participate in the conduction process, which grows nonlinearly with the electric field. 

According to the above analysis, the nonlinear conductance characteristics of CCTO/EDPM are related to the tunneling effect in high electric field. In a high electric field region, the relationship between the current density (J) and the applied electric field strength (E) should be satisfied by the following Equation (3):

J = Ae^(−B/E)^ (3)

**Figure 5:**
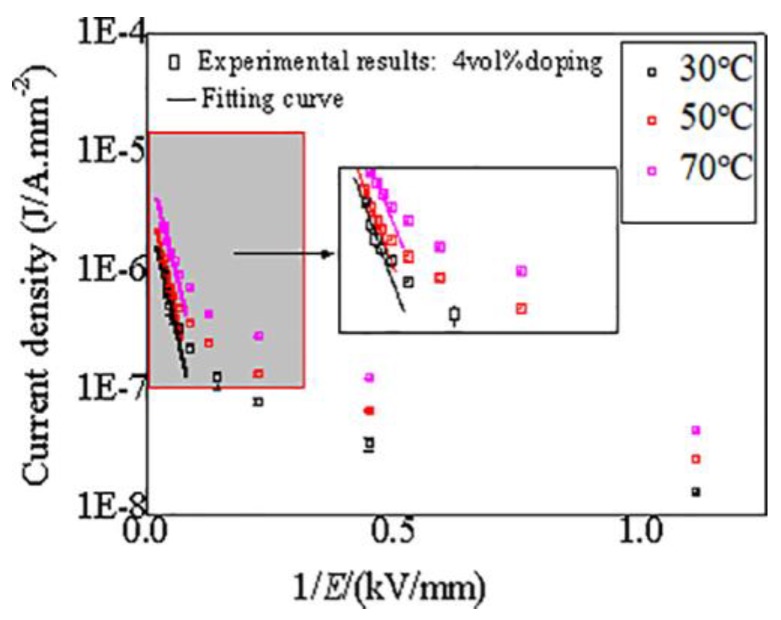
Fitted curves of tunneling effect in CCTO/EPDM composites at different temperatures.

**Figure 6:**
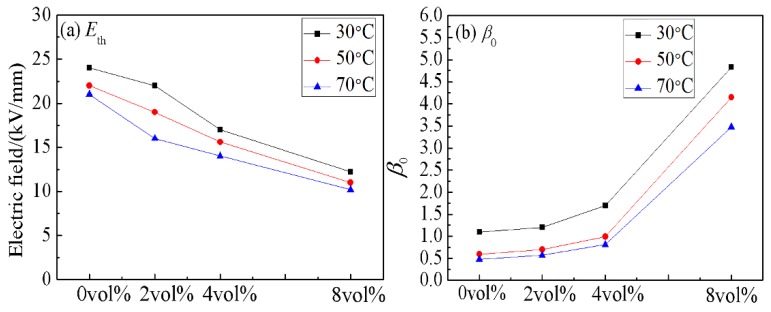
The nonlinear parameters of neat EPDM and CCTO/EDPM composites changes with temperature: (**a**) The threshold electric field E_th_; (**b**) The nonlinear coefficient β_0._

In Equation (3), the current density (J) is the ratio of measured current to measured area. A and B are constants, which relate to the work function between the electrode and medium, and show little relationship with temperature. In the high electric field area, lgJ and lgE should be linear in Equation (3). Figure 5 shows the tunneling fit curves of 4 vol % CCTO/EDPM at different temperatures, it can be seen that there is good linear relation between lgJ and lgE in high electric field area. This verifies that the conductance mechanisms of CCTO/EDPM composites are affected by the tunneling effect in areas of high electric field. With the exception of the E_th_ parameter, another important parameter to reflect the nonlinear characteristic is the nonlinear coefficient β_0_, defined as the ratio of lgγ to lgE under the E > E_th_, the nonlinear coefficient β_0_ changes with the increase of contents, as shown in Figure 6. It can be seen that the E_th_ decreases and β_0_ increases with increasing quantity of CCTO particles. Moreover, the 8 vol % CCTO/EPDM sample exhibits the best nonlinear conductivity and the lower threshold electric field (approximately 10 kV/mm). This is mainly because the average distance between particles decreases with increasing CCTO particle content in Figure 2, leading to the tunneling effect occurring at low electric field. In addition, based on the percolation theory [25], the higher the doping content, the easier the formation of the conductive path, leading to carriers transporting easily from cathode to anode, thus the nonlinear characteristics of CCTO/EPDM are more obvious.

#### 3.2.2. The Characteristic of Conductivity (γ)—Temperature (T)

Due to the structure and operation characteristics of the cable, there are both electric field gradients and temperature gradients in the insulation, so it is important to investigative the conductivity of CCTO/EPDM at different temperatures. According to the empirical formula, the relationship between the conductivity and temperature can be expressed as the following:

γ = γ_0_e^αT^ (4)

**Figure 7:**
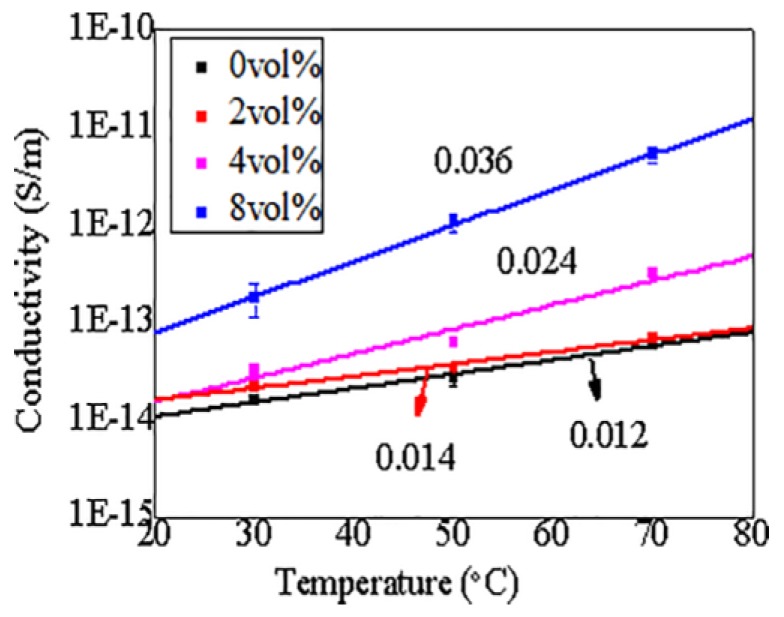
Relationship between conductivity and temperature characteristics of CCTO/EEPDM at 20 kV/mm.

In the Equation (4), α is the temperature coefficient and γ_0_ is a constant. Figure 7 shows the γ-T curve characteristics of CCTO/EPDM at high electric field (20 kV/mm). It can be seen that the temperature coefficient α of CCTO/EPDM is higher than that of neat EPDM, and that the conductivity of 8 vol % CCTO/EDPM composites increases by 2 orders of magnitude with the increase of temperature. It indicates that the conductivity of CCTO/EDPM is greatly affected by temperature, the conductivity of CCTO/EPDM increases significantly, it benefits reducing the difference in conductivity between cable accessories and XLPE insulation, caused by temperature changes, thereby improving the uneven electric field distribution. The conductivity of composites is related to nqμ (n is carrier density, q is charge amount of the carrier, and *μ* is carrier mobility), since the charge amount of the carrier is substantially constant, the conductivity of materials mainly depends on the carrier density and mobility. According to semiconductor physics, carrier mobility decreases with increasing temperature because of acoustic phonon scattering. Therefore, the reason for the increase of conductivity is not carrier mobility. Carrier density generally increase with increasing temperature due to thermal excitation, therefore the conductivity of composites increases with increasing temperature, leading to nonlinear conductivity that can be induced in a lower electric field, so the value of E_th_ reduces while temperature increases in Figure 6. Besides, the nonlinear conduction current density is related to eRE/kT (e is electron charge, R is average distance between local states, E is electric field, k is Boltzmann constant, and T is temperature), it can be seen that the nonlinear coefficient β_0_ decreases while temperature increases in Figure 6b, moreover it is related to the electric field.

### 3.3. The Dielectric Properties of Composites

**Figure 8:**
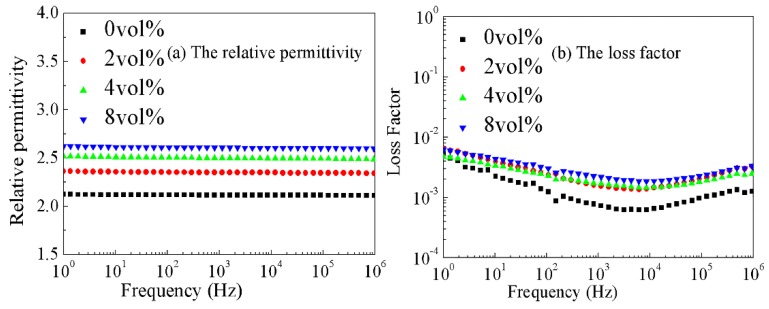
The relationship between frequencies and dielectric spectrum of EPDM rubber with different CCTO content: (**a**) The relative permittivity; (**b**) The loss factor.

Figure 8a shows the relationship between the frequency and relative permittivity of CCTO/EPDM composites. The results show that the permittivity of CCTO/EPDM composites increases with the increase of CCTO particles content, which depends on the dielectric polarization mechanism. The polarization effect is more obvious with CCTO particles with a higher relative permittivity [21,22]. Nelson et al. suggested that the doping of microsized fillers could decrease the free volume of composites, leading to relative permittivity increases [30]. The above complex mechanisms increase the relative permittivity. The permittivity of EPDM is 2.16, and the permittivity of 8 vol % CCTO/EPDM composites is 2.62.

Figure 8b shows the relationship between loss factor and frequency. The loss factor dropped first and then increased with frequency. Various polarizations have been established in low frequency, so the factor loss decreases while frequency increases. In the high frequency area, the relaxation polarization is too late to establish frequency increases, and the polarization of composites is mainly the displacement polarization, resulting in increased losses [30]. The loss factor increases with doping content of CCTO particles, which depends on conduction loss, mainly because the conductance loss play a major role ar this frequency range; the conductivity of CCTO/EPDM is higher than EPDM.

### 3.4. The Breakdown of Composites

**Figure 9:**
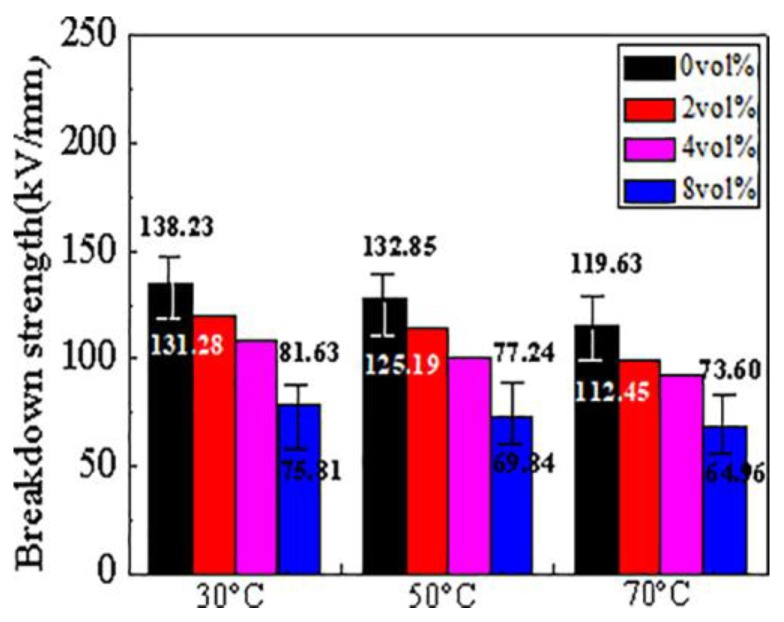
The breakdown strength of CCTO/EPDM composites.

It is well-known that the breakdown strength is important in nonlinear composites. The two-parameter Weibull distribution with 95% confidence intervals for CCTO/EDPM at different temperature is shown in Figure 9. It can be seen that the breakdown strength of CCTO/EPDM decreases with doping content of CCTO particles, and the breakdown strength of 8 vol % CCTO/EPDM is higher than 60 kV/mm at 70 °C, it still meets the needs of engineering [31]. Because CCTO particles are semiconductive, doping CCTO can introduce a large number of carriers, leading to the breakdown of composites [32,33]. Thus, the breakdown strength of CCTO/EPDM decreases with increasing CCTO particles content. Besides, the mobility of carriers increases with temperature, resulting in easier breakdown.

## 4. Simulation Results and Analysis of Electrical Field Distribution in DC Cable Termination

### 4.1. Distribution Characteristics of Steady Electrical Field in Cable Termination 

**Figure 10:**
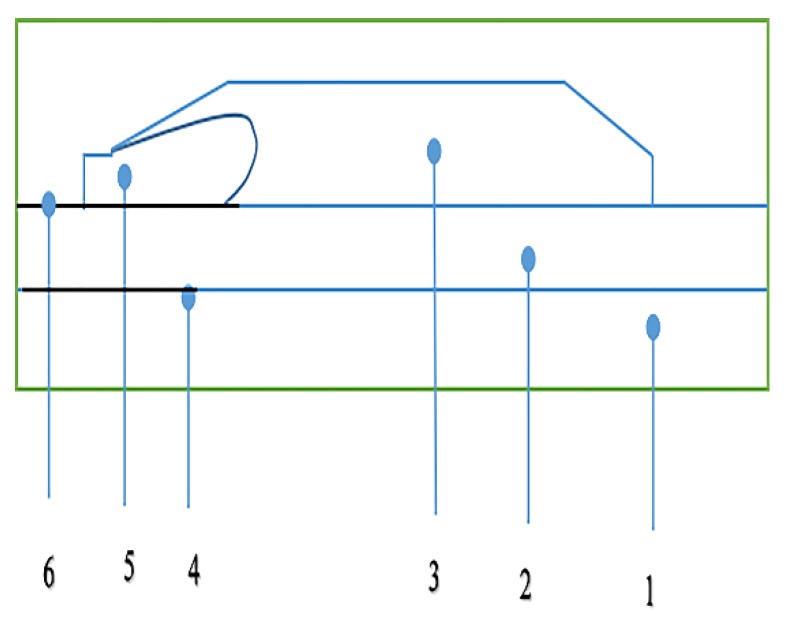
Cable termination model and simulation results: 1: core; 2: XLPE; 3: reinforced insulation; 4: inner shield; 5: stress cone; 6: outer shield.

**Figure 11:**
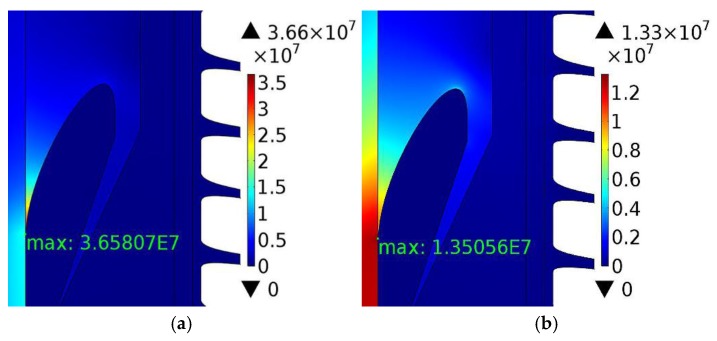
Cable termination simulation results: (**a**) The simulation results of EPDM; (**b**) the simulation results of 8 vol % CCTO/EPDM.

In order to analyze the ability of nonlinear conductive CCTO/EPDM composites for homogenizing the electric field, the COMSOL Multiphysics simulation was employed. The core voltage (U_0_) was 200 kV and the core temperature was 70 °C. The schematic of cable termination can be seen in Figure 10. Figure 11 shows the simulation results of the electric field distribution in cable termination during the DC steady state. It can be found that 8 vol % CCTO/EPDM can make the electrical field evenly distributed in cable termination, and the maximum electrical field of the root is 1.35 × 10^7^ V/m, it does not exceed the DC breakdown strength of CCTO/EPDM. 

### 4.2. Distribution Characteristics of Transient Electrical Field in Cable Termination

There are transient processes, such as polarity reversal, and a lightning impulse process in the course of HVDC transmission. Transient processes are more easily to aggravate the destruction of the cable system. Aiming at the problem of electric field distribution in HVDC cable insulation, some researchers used simulation software to study the steady-state electric field distribution in cable insulation under a temperature gradient [34]. However, there is no relevant research report on the transient electric field distribution of DC cable insulation. Thus, this section mainly investigates the influence of lightning impulse voltage and polarity reversal on the electric field distribution of cable termination, especially at the root of the stress cone.

**Figure 12:**
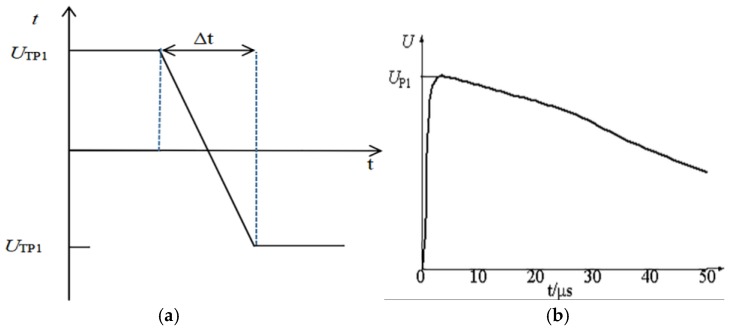
Waveform of applied voltage during the transient process: (**a**) Polarity reversal process applied voltage waveform; (**b**) lightning impulse voltage waveform.

In the process of polarity reversal, external applied voltage amplitude U_TP1_ = 1.45U_0_. The waveform of the applied voltage is shown in Figure 12a during the polarity reversal, Δt is polarity reversal time. When the applied voltage is reversed from the negative polarity to the positive polarity, the distribution characteristics of the electrical field are not substantially changed. Therefore, only the polarity reversal process of the positive polarity to the negative polarity is studied. Before the polarity reversal occurs, the cable termination is a steady operation. The waveform of lightning impulse process is shown in Figure 12b. GB/T 3048.13-2007, taking a standard 1.2/50 μs lighting impulse voltage waveform. In the lightning impulse process the applied voltage amplitude U_P1_=1050 kV [35]. In this section, 8 vol % CCTO/EPDM replaces traditional reinforced insulation. 

#### 4.2.1. The Electric Field Distribution of Cable Termination during Polarity Reversal

**Figure 13:**
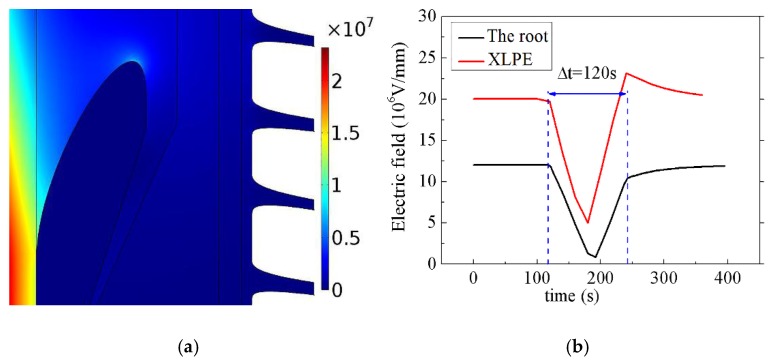
Electric field distribution inside the cable termination in the polarity reversal process: (**a**) When the applied voltage reaches-U_TP1_; (**b**) variation of the electric field strength with respect to time.

8 vol % CCTO/EPDM composites are replaced with traditional reinforced insulation materials. Set the applied external applied voltage U_TP1_ = 1.45U_0_, Δt = 120 s, the variation of the field strength of XLPE and the root in polarity reversal process is shown in Figure 13a. Firstly, when the applied voltage reaches -U_TP1_, the maximum electrical field is 2.37 × 10^7^ V/m, concentrated in XLPE, which is higher than the root. Secondly, when the polarity reversal process ends, and the electric field reaches -U_TP1_, the electric field strength of XPLE is significantly higher than before the polarity reversal, and the electric field of the root is opposite in Figure 13b. The reason is the presence of a different charge in the insulator after polarity reversal, resulting in the increase of the maximum electric field in XLPE insulation. This kind of different polarity charge is different from the space charge determined by the trapping factor under the uniform electric field. It is determined by the slow polarization, which is caused by the nonlinearity of the material. The charge is the same polarity charge before polarity reversal, and the charge is the different polarity after polarity reversal. The charge of the root may be opposite to the above condition [34].

#### 4.2.2. The Electric Field Distribution of Cable Termination during Lightning Process

Overhead lines are more likely to be affected by lightning, resulting in higher possibility of line trips and faults. Due to the fact that cables are connected with overhead lines, this will also be affected by the lightning impulse voltage. This section investigates the characteristics of the electrical field distribution in the cable terminal when the working voltage of the HVDC cable is superimposed with a positive and negative lightning pulse, respectively. The lightning impulse waveform is shown in Figure 12b. 

**Figure 14:**
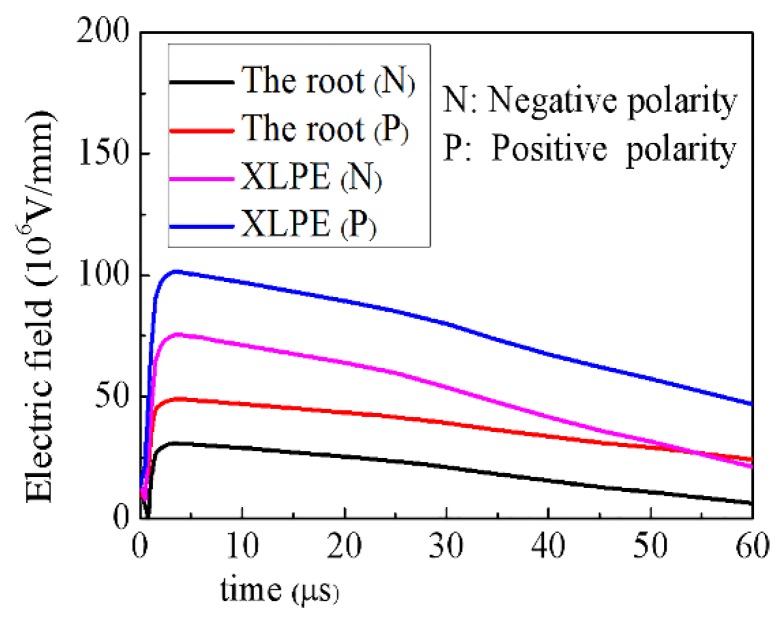
Variation of the electric field strength with respect to time in the lightning pulse process.

**Figure 15:**
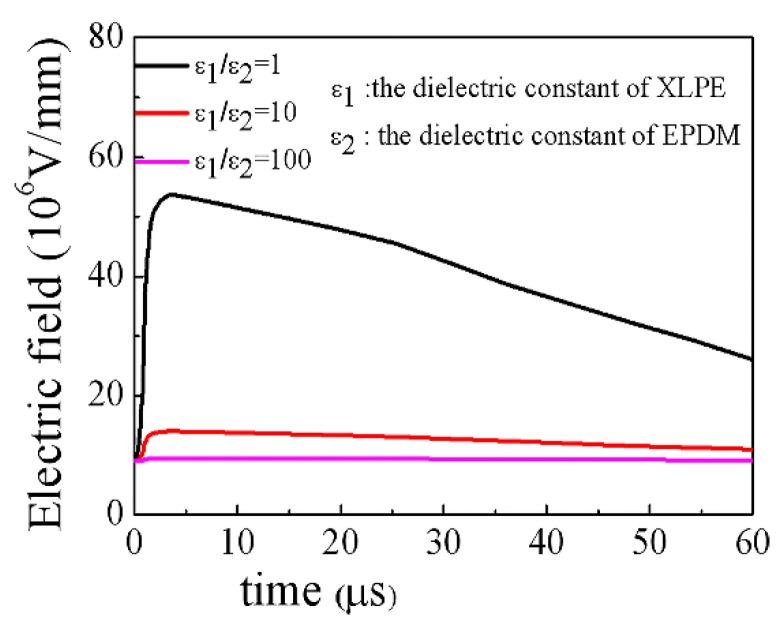
Variation of the electric field strength with different dielectric constants in the lightning impulse process.

Figure 14 shows the relationship between the maximum field strength of XLPE and root with time under different polarity lightning pulse. The electric field of the root is always less than the electric field of XLPE in termination, it does not change with the polarity of the lightning impulse voltage, and the electric field strength of the root is much lower than the breakdown strength. This is due to fact that the conductivity of composites no longer plays a dominant role in the distribution of the electric field in cable termination during the lightning pulse process and that the dielectric constant of composites also participates in determining the electric field distribution. Figure 15 shows the influence of the dielectric constant on the distribution of the electric field in termination during a positive lightning impulse. The simulation results show that the increase of the dielectric constant can significantly reduce the maximum electric field at the root in lightning impulse process. Therefore, the development of composites with both nonlinear conductance and dielectric properties will be the focus of research on DC cable accessories.

## 5. Conclusions

This paper mainly studies the influence of CCTO particle content on the nonlinear conductivity of CCTO/EPDM composites; it also analyzes the formation reasons of nonlinear conductivity, and studies the distribution of electric fields under steady state and transient conditions, respectively. The conclusion follows:

(1) The CCTO particles are fabricated by the sol-gel method, and dispersed evenly in CCTO/EPDM composites. CCTO/EPDM composites have good nonlinear conductance characteristics. When the content of CCTO particles is low, the conductivity of CCTO/EDPM does not exhibit nonlinear characteristics; with the increase of the doping content, the conductivity of CCTO/EDPM shows a nonlinear increase with the increase of the electric field strength. Moreover, the E_th_ reduces and β increases with the increase of CCTO particle content.
(2) The increase of CCTO particle content, leading to the free volume decreases; and due to the high dielectric constant of CCTO particles, leading to the dielectric constant of CCTO/EDPM composites increases with the increase of CCTO particles content.
(3) The simulation results of steady and transient electric field distributions show that CCTO/EPDM composites exhibit ability to homogenize the electric field distribution.
(4) In the lightning impulse process, the maximum electric field is concentrated on XLPE, and it does not change with the polarity of the lightning pulse process. The dielectric constant increase of reinforced insulation can significantly reduce the maximum electric field strength of the root.

## Figures and Tables

**Table 1:** List of drugs for this experiment.

Materials	Corporation
Ca(NO_3_)_2_·4H_2_O (≥ 99%)	Sinopharm Chemical Reagent Co., Ltd., Shanghai, China
Cu(NO_3_)_2_·3H_2_O (≥ 99%)	
Ti(OC_4_H_9_)_4_ (≥ 99%)	
C_3_H_8_O_2_ (≥ 99%)	
